# Does pain relate with activation of quadriceps and hamstrings muscles during strengthening exercise in people with knee osteoarthritis?

**DOI:** 10.1186/s40064-016-2048-1

**Published:** 2016-04-14

**Authors:** Elora C. Brenneman, Alexander B. Kuntz, Emily G. Wiebenga, Monica R. Maly

**Affiliations:** School of Rehabilitation Science, McMaster University, 1400 Main Street West, Hamilton, ON L8S 1C7 Canada; Department of Kinesiology, McMaster University, 1280 Main Street West, Hamilton, ON L8S 4L8 Canada

**Keywords:** Arthritis, Biomechanics, Musculoskeletal pain, Resistance training, Osteoarthrosis

## Abstract

Muscle strengthening may be difficult to achieve in knee osteoarthritis (OA) due to pain. A large knee adduction moment (KAM), representing medial relative to lateral knee load, may also relate with pain during strengthening exercise. The objective of this study was to examine relationships between knee pain status and electromyography (EMG) amplitude of knee muscles during squat and lunge exercises. We also evaluated relationships between pain and KAM during these exercises. Forty-two women with symptomatic knee OA participated. Knee pain intensity and frequency were captured with two reliable and valid questionnaires. Motion analyses of squat and lunge exercises were completed. Total average EMG amplitude across five muscles of the lower limb and average KAM were calculated from the static portion of these exercises. Multiple regression analyses examined the relationships between pain and total average EMG amplitude; and pain and average KAM during squats and lunges. Pain improved the model for KAM from the trailing leg of a lunge. Pain did not improve any other model. Overall, pain may not be a useful indicator of EMG amplitude or KAM during exercise in knee OA.

## Background

Strengthening exercise is the cornerstone of conservative treatment for knee osteoarthritis (OA) (Bennell et al. 2008). Yet, strengthening exercise programs have a small effect size in improving strength in knee OA (Fransen and McConnell 2008; Bennell et al. 2013). It is possible that this small effect size occurs because pain impairs muscle activation, therefore limiting the capacity for strengthening in knee OA. It is also possible that pain reflects an altered loading environment at the knee that is potentially damaging. As a result, achieving meaningful improvements in leg muscle strength through exercise may be compromised by the presence of pain in people with knee osteoarthritis (OA).

People with chronic knee pain have reduced quadriceps strength and difficulty voluntarily activating the quadriceps compared to pain-free individuals (Bennell et al. 2008, 2013; O’Reilly et al. 1998). Pain reduces physical activity (Holla et al. 2014) and is thought to reduce the ability to voluntarily activate muscle (O’Reilly et al. 1998; Berth et al. 2002). The latter—arthrogenic inhibition—explained the greatest amount of total variance in quadriceps strength deficit following total knee replacement for severe knee OA (Stevens et al. 2003). It has been suggested that people with knee OA may correct inhibition with exercise training (Lewek et al. 2004). However, the opposite has also been observed, where data from 40 participants with mild to moderate knee OA showed that pain was related to greater electromyography (EMG) amplitude and duration in knee OA (Astephen Wilson et al. 2011). Finally, a recent study discovered that pain did not influence thigh muscle EMG amplitudes or proprioceptive acuity in people with mild and moderate knee OA (n = 31) during a stair climbing task (de Oliveira et al. 2014). These disparate findings show that the relationships of pain and muscle activation during strengthening exercise remain unclear in knee OA. Clarity of this relationship is important because clinicians are challenged to improve pain through exercise; but may also facilitate arthrogenic inhibition by inducing pain with exercise.

Pain likely also has an impact on the loading environment at the knee in those with OA. During gait, both pain intensity and the knee adduction moment (KAM), a measure representing the distribution of medial versus lateral loading at the knee joint, change in response to administration of oral or injectable pain medication (Schnitzer et al. 1993; De et al. 2000). Use of non-steroidal anti-inflammatories concurrently reduced pain and increased the adduction and quadriceps moments, suggesting that pain may be a mechanism by which people with knee OA modify their gait in an effort to decrease the KAM (Schnitzer et al. 1993). This relationship between pain and KAM may be influenced by an increase in gait velocity (Robbins and Maly 2009). A positive relationship between peak KAM and walking velocity, and a negative relationship between pain and walking velocity, were observed in people with moderate to severe radiographic knee OA (Henriksen et al. 2012). Exploring these variables during static weight-bearing exercises may provide clarity into whether pain and KAM relate, without the influence of speed of movement.

The purpose of this investigation was to identify the relationships of pain intensity and frequency with total average EMG amplitude of five muscles crossing the knee during static squat and lunge exercises in women with symptomatic knee OA. A secondary purpose was to evaluate the relationships of pain with average KAM during squats and lunges. We hypothesized that greater pain intensity and frequency would relate with greater total average EMG amplitude of knee muscles during squats and lunges; and pain would not relate with KAM during squats and lunges in women with symptomatic knee OA. This knowledge will directly contribute to future work to determine the appropriate type of exercise for people with painful knee osteoarthritis that stimulates lower limb musculature to improve strength and promotes an optimal loading environment at the knee joint.

## Results

Mean (standard deviation) age and body mass index of the 42 participants were 60.7 (6.5) years and 29.6 (5.6) kg/m^2^, respectively. Table 1 provides descriptive statistics for the independent (pain) and dependent (total average EMG amplitudes and KAM) measures and Table 2 outlines individual muscle EMG amplitudes during each exercise.

**Table 1:** Mean (standard deviation) values for the independent variables and dependent variables among 42 women with symptomatic knee OA

	Mean (standard deviation)	Minimum, maximum
*Independent variables*		
KOOS pain score (/100)^a^	67.1 (14.9)	25.0, 92.0
ICOAP total score (/100)^b^	31.3 (20.2)	0.0, 93.2
*Dependent variables*		
Total average EMG amplitude (%MVIC)		
Legs-together squat^ɸ^	18.5 (6.8)	8.1, 33.3
Wide-legged squat^Γ^	17.8 (6.8)	5.0, 34.8
Lunge (leading leg)^Γ^	15.8 (6.1)	7.5, 31.7
Lunge (trailing leg)^ɸ^	14.0 (5.4)	6.0, 28.1
KAM (Nm/kg)		
Legs-together squat	−0.04 (0.13)	−0.48, 0.34
Wide-legged squat	−0.02 (0.14)	−0.36, 0.28
Lunge (leading leg)	0.08 (0.13)	−0.22, 0.38
Lunge (trailing leg)	−0.08 (0.14)	−0.29, 0.33

*KOOS* Knee Injury and Osteoarthritis Outcome Score, *ICOAP* Intermittent and Constant Osteoarthritis Pain questionnaire,  %*MVIC*  % maximum voluntary isometric contraction, *KAM* knee adduction moment

^a^Higher scores indicate less pain

^b^Higher scores indicate more pain

^ɸ^7 cases where 4 muscles were averaged

^Γ^8 cases where 4 muscles were averaged

**Table 2:** Mean (standard deviation) amplitude of individual muscle demands during the four exercises

	Biceps femoris^a^	Semitendinosus^b^	Rectus femoris	Vastus lateralis^c^	Vastus medialis^c^
Legs-together squat	12.1 (7.3)	10.0 (6.5)	18.9 (9.6)	29.0 (12.4)	21.4 (13.0)
Wide-legged squat	11.3 (6.5)	10.1 (6.6)	18.3 (9.5)	25.8 (13.1)	22.7 (12.4)
Lunge (leading leg)	13.4 (7.6)	10.4 (6.7)	14.7 (7.9)	21.8 (11.7)	18.8 (10.5)
Lunge (trailing leg)	11.7 (7.1)	9.9 (6.6)	14.4 (7.1)	17.3 (9.2)	16.3 (9.3)

Note that for the vastus lateralis muscle only the Wide-legged squat had a missing case

^a^Missing cases n = 2

^b^Missing cases n = 4

^c^Missing cases n = 1

Table 3 summarizes the models of pain and total average EMG amplitude during squat and lunge exercises. No measures of pain improved model variance prediction for the exercises analyzed.

**Table 3:** Multiple linear regressions for total average EMG amplitude for each of the four exercises

Dependent	Independent	R-square	Unstandardized β coefficient	Standardized β coefficient	Change statistics p (df, F)
Legs-together squat (%MVIC)	KOOS pain	0.055	−.107	−.235	0.134 (40, 2.339)
	ICOAP total	0.061	0.083	0.247	0.115 (40, 2.598)
	NRPS	0.000	0.067	0.021	0.894 (40, 0.018)
Wide-legged squat (%MVIC)	KOOS pain	0.014	−.053	−.116	0.464 (40, 0.548)
	ICOAP total	0.013	0.038	0.113	0.474 (40, 0.522)
	NPRS	0.002	−.124	−.039	0.806 (40, 0.061)
Lunge (leading leg) (%MVIC)	KOOS pain	0.055	−.096	−.235	0.134 (40, 2.344)
	ICOAP total	0.055	0.071	0.235	0.134 (40, 2.344)
	NPRS	0.003	−.155	−.055	0.730 (40, 0.121)
Lunge (trailing leg) (%MVIC)	KOOS pain	0.022	−.053	−.148	0.349 (40, 0.897)
	ICOAP total	0.008	0.024	0.091	0.565 (40, 0.337)
	NPRS	0.000	0.054	0.022	0.892 (40, 0.019)

A summary of multiple linear regressions of mean KAM during lunge and squat exercises and pain measures is included in Table 4. KOOS pain scale and ICOAP total score each explained variance for the trailing leg in a lunge (R-squared = 0.108, p = 0.033; R-squared = 0.115, p = 0.028 respectively). Pain did not improve total explained variance of the model for the legs-together squat (R-square ≥ 0.013, p ≥ 0.349), the wide-legged squat (R-square ≥ 0.002, p ≥ 0.262), or the leading leg in the lunge pose (R-square ≥ 0.003, p ≥ 0.589).

**Table 4:** Multiple linear regression analyses for mean KAM during the squat and lunge exercises

Dependent	Independent	R-square	Unstandardized β coefficient	Standardized β coefficient	Change statistics p (df, F)
Legs-together squat (Nm/kg)	KOOS pain	0.013	−.001	−.115	0.467 (40, 0.540)
	ICOAP total	0.022	0.001	0.148	0.349 (40, 0.898)
	NPRS	0.019	−.009	−.140	0.378 (40, 0.794)
Wide-legged squat (Nm/kg)	KOOS pain	0.004	−.001	−.060	0.707 (40, 0.143)
	ICOAP total	0.031	0.001	0.177	0.262 (40, 1.293)
	NPRS	0.002	−.003	−.047	0.767 (40, 0.089)
Lunge (leading leg) (Nm/kg)	KOOS pain	0.007	0.001	0.086	0.589 (40, 0.297)
	ICOAP total	0.003	0.000	−.058	0.716 (40, 0.134)
	NPRS	0.004	−.004	−.066	0.680 (40, 0.173)
Lunge (trailing leg) (Nm/kg)	KOOS pain	0.108	−.003	−.329	0.033* (40, 4.864)
	ICOAP total	0.115	0.002	0.339	0.028* (40, 5.201)
	NPRS	0.005	−.004	−.067	0.672 (40, 0.182)

Independent variables included measures of pain

* Significance (α < 0.05)

## Discussion

This study examined relationships of self-reported pain with daily activities, as well as pain experiences during maximal isometric efforts, with total average EMG amplitude during static exercises in women with symptomatic knee OA. Pain did not explain variance for total average EMG amplitude of quadriceps and hamstrings muscles. This finding suggests that chronic pain and pain experiences during maximal isometric contractions are not closely linked with total average EMG amplitude in women with knee OA during static exercise. Our second purpose was to identify the influence of pain on mean KAM during static exercises. The KOOS pain subscale (pain experienced over 1 week), and ICOAP total score (pain intensity and frequency) explained variance of KAM in the trailing leg of a lunge. Otherwise, pain measures did not relate with mean KAM of the leading leg lunge or either squat task.

Our results are consistent with previous work that demonstrated pain had little relationship with loss of voluntary quadriceps amplitude prior-to and 1 month following knee arthroplasty (Mizner et al. 2005). Also consistent with our findings, pain, though a statistically significant contributor, was not the most important predictor of central activation ratio in 28 patients with unilateral end-stage primary knee OA that were tested 10 days before and 26 days after knee arthroplasty (Stevens et al. 2003). From a clinical perspective, these data suggest that the presence of pain is likely not a factor that will limit the ability to activate muscles around the knee during strengthening exercise among people with knee OA.

While studies directly relating measures of pain with EMG amplitude in knee OA are scarce, there is some controversy. It has been suggested that a decrease in activation occurs due to peripheral inhibition (Berth et al. 2002). However, the opposite has also been suggested by a group that examined the relationship between pain and EMG activation in 40 people with clinical knee OA (Astephen Wilson et al. 2011). Greater pain intensity was associated with increased EMG amplitude and activation duration of the medial hamstrings during gait. This increased activation may have reflected a pattern aimed at balancing muscle forces across a damaged joint (Astephen Wilson et al. 2011). As well, pain did not affect either proprioceptive acuity or thigh muscle EMG amplitudes during a stair climbing task in people with mild to moderate knee OA (de Oliveira et al. 2014). Thus, it remains unclear whether the presence of pain increases or decreases in muscle amplitudes of the quadriceps and hamstrings in people with knee OA. Given the data from the current study, we suggest that inhibition across all major knee joint muscles does not occur.

Pain is not an indication of the distribution of medial versus lateral joint loading in people with knee OA during static exercises. The KAM is a useful indicator of disease progression and severity in knee OA (Sharma et al. 1998; Miyazaki et al. 2002; Bennell et al. 2011). However, an inconsistent relationship appears between pain and KAM. An analgesic-induced decrease in pain produces an increase in peak KAM during gait (Schnitzer et al. 1993; De et al. 2000; Robbins and Maly 2009). Cross-sectional studies offer less consistency. Studies have demonstrated greater peak KAM during gait in symptomatic versus asymptomatic radiographic matched controls (Henriksen et al. 2012), an inverse association between pain and late-stance KAM in women with non-radiographic knee pain [β = −10.1 95 % CI (−17.6, −2.7), p = 0.01] (Thorp et al. 2007), and an inverse association between pain and KAM in less severe knee OA (K/L grade ≤2) [slope (SE) = −0.101 (0.059), p = 0.008] (Teichtahl et al. 2006). Meanwhile other studies show no relationship between KAM and pain (Hurwitz et al. 2002; Maly et al. 2008). Inconsistency in pain questionnaires used and variations in pain experience between individuals may account for differences observed. As well, gait velocity may influence the relationship between pain and peak KAM. Pain and peak KAM were obtained from people with radiographically mild (K/L ≤ 2; n = 68) and radiographically severe (K/L > 2; n = 69) medial knee OA (Henriksen et al. 2012). Significant negative relationships between pain and gait velocity and between peak KAM and gait were observed in both groups (p = 0.047 and p < 0.001 respectively). Because a cross-sectional assessment of pain offers little insight into KAM, pain cannot guide exercise prescription if the goal is to minimize the medial versus lateral knee loading implicated in knee OA progression (Mizner et al. 2005; Teichtahl et al. 2006; Hurwitz et al. 2002).

This study had limitations. First, several relationships were computed which increased the likelihood of finding significance by chance. The findings from this study identify that the relationships of pain and EMG amplitude, or pain and KAM, are poor. It is possible that a greater number of study participants would have improved the correlations noted between pain and EMG amplitude, or pain and KAM. However, each regression analysis followed the rule of 10 participants per predictive variable (Zar 1999) and the sample size was adequate based on the sample size estimate calculated for this study. Given the inconsistencies in these relationships reported in the literature, the relationships of pain and EMG amplitude and pain and KAM are likely poor at best. Second, pain was not recorded after the weight-bearing exercises. Third, it is possible that true MVICs may not have been attained from the lower limb muscles on the dynamometer. However, twitch potentiation, the gold standard of achieving of maximal activation, is invasive and painful. Fourth, selection bias and generalizability error may be present in the sample. The dataset was extracted from baseline data of a single-cohort exercise intervention study and therefore those that are more mobile or experience less pain overall were more likely to participate. Finally, there was no control group in the original study.

## Conclusions

In summary, among women with symptomatic knee OA, cross-sectional relationships between pain and EMG amplitude during squatting and lunging exercises were poor. Further, pain was not a useful indicator of KAM during exercise. In the prescription of static squat and lunge exercises, pain likely does not interfere with efforts to strengthen the knee musculature. Pain is also not recommended to provide insight as to whether exercise overloads the damaged knee with OA. Future work should aim to investigate frequency and duration of these static weight-bearing exercise on strengthening, joint loading, and measures of pain.

## Methods

This study was a secondary, cross-sectional analysis of baseline data collected for an intervention study (Clinical Trial registration number: NCT02146105) (Brenneman et al. 2015).

Participants were recruited through rheumatology, orthopaedic, and physical therapy clinics, as well as by word-of-mouth and social media. All participants were screened by a rheumatologist and/or trained research assistant. Forty-two community dwelling older women with symptomatic knee OA participated. All met the criteria for clinical knee OA according to the American College of Rheumatology (ACR) (Altman et al. 1986). These clinical ACR criteria include being 50 years of age or older and answering “yes” to three of the following six criteria: having knee pain on most days of the week, crepitus, bony tenderness or enlargement, inflammation, or morning knee stiffness lasting longer than 30 min (Altman et al. 1986). Exclusion criteria included other forms of arthritis, knee surgery, lower limb trauma in the last 3 months, use of an assistive walking aid, ipsilateral hip or ankle conditions, pregnancy, or health conditions that may be exacerbated by the protocol. This study was approved through the institutional ethics board and all participants provided written, informed consent.

An estimation of sample size was completed a priori using a *t* test for mean difference from a constant (one sample case) (Faul et al. 2007). A conservative effect size of 0.5 [the effect of resistance exercise on self-reported outcomes have yielded effect sizes up to 2.11 in the literature (Lange et al. 2008)] and a Type 1 error of 0.05 on a two-tailed test were assumed. A minimum of 34 participants were required to yield 80 % power capable of detecting significant change.

The most symptomatic knee was selected for measurements. The most symptomatic knee refers to the knee self-reported to experience greater intensity of symptoms. Knee pain status was recorded using three scales to capture aspects of the multidimensionality of pain. First, self-reported pain intensity typically experienced over the past 7 days was captured using the Knee injury and Osteoarthritis Outcome Score (KOOS) pain subscale. This subscale rates knee pain intensity on a five-point Likert scale (0 = no pain, 4 = intense pain) during nine activities. The KOOS pain subscale has a high test–retest reliability with intra-class correlation coefficients of 0.80–0.97 (Alviar et al. 2011). It also produces valid measurements in knee OA, with Cronbach’s alpha values ranging from 0.65 to 0.94 (Collins et al. 2011). A score from the KOOS pain subscale was calculated as a normalized score out of 100 (KOOS Scoring 2012) where higher scores reflect less pain (Roos et al. 1998).

Second, pain frequency during daily living was reflected by the Intermittent and Constant Osteoarthritis Pain (ICOAP) questionnaire. The ICOAP reflects intermittent and constant symptoms of their most troublesome knee, and includes questions regarding pain intensity, frequency, and impact on mood, sleep, and quality of life in the last week. Questions are scored on a 5-point Likert scale (0 = no pain, 4 = intense pain) with higher overall scores indicating more pain. The ICOAP produces reliable (ICC = 0.85) (Bombardier et al. 2011) and valid (Cronbach’s alpha = 0.93) (Hawker et al. 2008) data in knee OA. The total score for the ICOAP was calculated and normalized out of 100.

Third, ratings of knee pain intensity with muscle activity were collected using the Numeric Pain Rating Scale (NPRS) immediately following a maximum effort knee extensor contraction. The NPRS produces reliable (Stratford and Spadoni 2001) and valid data (Downie et al. 1978) in people with knee OA. Following knee extensions, the participants rated their knee pain between 0 and 10 (0 = no pain, 10 = worst pain imaginable).

Electromyography and motion analyses of static squat and lunge exercises were completed to yield EMG amplitudes (expressed relative to a maximum voluntary isometric contraction) and KAM values. Squat and lunge exercises were chosen because these are commonly recommended, functional exercises that require little equipment (Bennell et al. 2011; Ageberg et al. 2013). During these exercises, EMG activations were recorded from quadriceps and hamstring muscles. Mean KAM was determined for static exercises and was expressed relative to body mass (Nm/kg) to enable comparisons between study participants. Details regarding acquisition and processing of these data are provided below.

Electromyography signals were collected with a wire-minimal EMG sensor (dual differential amplifier, CMRR > 100 dB, input impedance >100 MΩ). These EMG signals were sampled at 1500 Hz (Noraxon U.S.A. Inc., Scottsdale, AZ). Participants were instrumented with five Ag–AgCl dual electrodes (Natus Neurology Inc., Middleton, WI) on each of biceps femoris, semitendinosus, rectus femoris, vastus lateralis, and vastus medialis using procedures consistent with Surface Electromyography for the Non-Invasive Assessment of Muscles guidelines (www.seniam.org, Enschede, Netherlands) (Hermens et al. 2000). These EMG data were normalized to maximum voluntary isometric contractions (MVIC). After a submaximal warm-up, participants completed five MVICs of the knee extensors and flexors with the knee positioned in 65^o^ of flexion on a dynamometer (Biodex Medical Systems, Inc., Shirley, NY, USA). Maximum activation was determined as the peak activation in each muscle from any of the MVIC contractions.

Synchronized with EMG, motion analyses were conducted. Each participant was instrumented with four skin-mounted motion capture marker clusters placed on the foot, shank, and thigh and sacrum. Each marker cluster was outfitted with three infrared-emitting diodes arranged in a triangular orientation. Virtual markers were identified during a static standing trial to define anatomical coordinate systems using a digitizer outfitted with markers. Three-dimensional motion of the clusters was collected with a nine-camera system (Optotrak Certus, Northern Digital Inc., Waterloo, ON) at 100 Hz. Simultaneously, ground reaction forces were captured at 1000 Hz with floor-embedded force plates (Advanced Mechanical Technologies Inc., Watertown, MA).

**Fig. 1:**
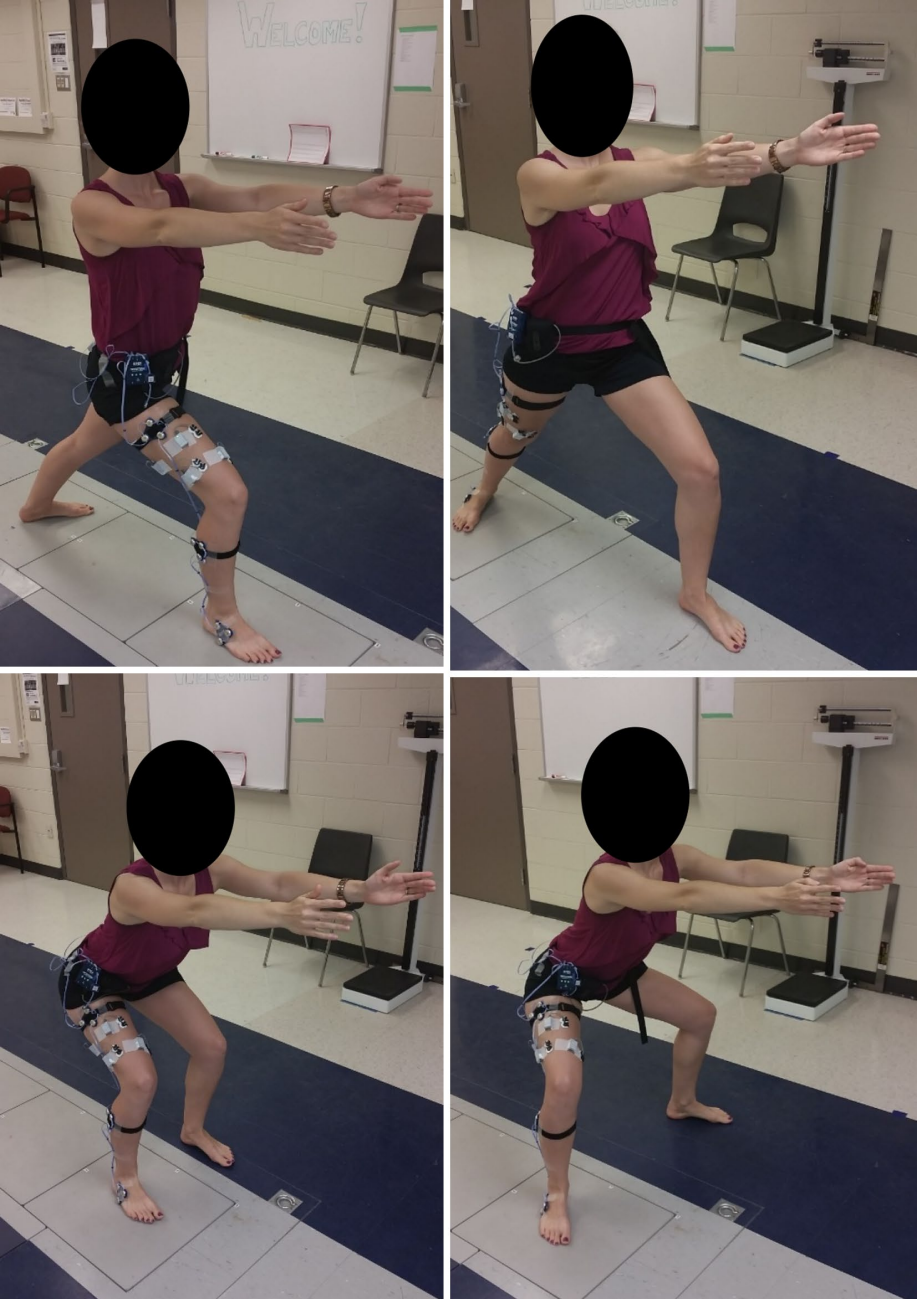
Four exercises completed by participants: two variations of a double-legged squat (*top*) and two lunge poses with alternating legs as the leading leg (*bottom*). All participants were asked to keep their instrumented leg, the most symptomatic knee, on the force plate throughout the task

Once instrumented, participants completed four exercises barefoot. Participants completed two variations of a squat and two variations of a lunge (Fig. 1). The squat variations included a “legs-together” squat (feet hip width apart) and a wide-legged squat (feet greater than shoulder width apart and externally rotated). In both, participants were instructed to squat while aligning their knees with the 2^nd^ digit of the foot in the sagittal plane. Participants were asked to keep their back neutral and to flex the shoulders to 90°, with elbows fully extended. Participants were also given the instruction to lead with the hips as if they were going to sit back in a chair behind them. This allowed their knees to flex without positioning the knees anterior to the toes.

Lunge variations were completed twice: first with the most symptomatic knee as the leading leg and second with the most symptomatic knee as the trailing leg. The lunges involved a staggered stance with the front knee flexed (leading leg) and the opposite leg in hip and knee extension with the foot planted on the ground (trailing leg). Participants were asked to maintain the hips in the coronal plane as much as possible, flex the leading knee in line with the 2nd digit of the foot, and flex the shoulders to 90° with elbows fully extended. Similar instructions on knee position were given in the lunge tasks as in the squat tasks, where the knee was not to be positioned anterior to the toes. Exercises were held statically for 10 s and repeated three times.

The KAM waveform was calculated using inverse dynamics (C-Motion Inc., Kingston, ON) (Winter 2009). Kinematic and kinetic data were filtered using a 6 Hz dual pass Butterworth filter to minimize signal artefact. All EMG data were zeroed, full-wave rectified, linear-enveloped using a second order low pass Butterworth filter with a cut-off of 3 Hz, and normalized to %MVIC (Mathworks, Inc., Natick, MA). Total average EMG amplitude was calculated for each exercise by summating the %MVIC values of each muscle and dividing by the total number of muscles (five). Total average EMG amplitude of muscles crossing the knee was used in lieu of EMG amplitudes for individual muscles to provide an overall indication of the response to joint demand. Because EMG amplitude was recorded from three quadriceps muscles and two hamstrings muscles, the total average EMG amplitude places greater emphasis on the quadriceps. The squat and lunge tasks investigated are targeting strengthening in the quadriceps group, a milestone in knee OA management (Bennell et al. 2013). Using this measure of overall joint demand eliminates the potential for conducting multiple statistical analyses for individual muscles.

To calculate EMG and KAM measures during the exercises, a moving average of the coefficient of variation of the KAM was calculated throughout the static activity (values within ± three standard deviations of the mean KAM during the middle second). A three-second window of the lowest coefficient of variation was chosen for analysis of average EMG amplitude and average KAM. These variables were ensemble averaged across two successful trials.

Descriptive statistics were calculated for the sample. To test the first hypothesis that greater pain intensity and frequency would relate with greater total average EMG amplitude, multiple linear regression models were used with total average EMG amplitude during squat and lunge exercises as the dependent variable; and each pain measure (KOOS pain, ICOAP total score, and NPRS) as separate independent variables. To test the second hypothesis that pain would not relate with KAM during squats and lunges, multiple linear regression models were used. The dependent variables included mean KAM during each of the squat and lunge exercises and independent variables were each pain measure. All statistical tests were completed in SPSS software (IBM, Inc., Version 21).
